# The cooperative function of arginine residues in the Prototype Foamy Virus Gag C-terminus mediates viral and cellular RNA encapsidation

**DOI:** 10.1186/s12977-014-0087-7

**Published:** 2014-10-08

**Authors:** Martin V Hamann, Erik Müllers, Juliane Reh, Nicole Stanke, Gregory Effantin, Winfried Weissenhorn, Dirk Lindemann

**Affiliations:** Institute of Virology, Medical Faculty “Carl Gustav Carus”, Technische Universität Dresden, Fetscherstr. 74, 01307 Dresden, Germany; CRTD/DFG-Center for Regenerative Therapies Dresden - Cluster of Excellence, Technische Universität Dresden, Fetscherstr. 105, 01307 Dresden, Germany; Univ. Grenoble Alpes, UVHCI, F-38000 Grenoble, France; CNRS, UVHCI, F-38000 Grenoble, France; Present address: Department of Cell and Molecular Biology, Karolinska Institutet, 171 77 Stockholm, Sweden

**Keywords:** Foamy virus, RNA packaging, Gag, Assembly

## Abstract

**Background:**

One unique feature of the foamy virus (FV) capsid protein Gag is the absence of Cys-His motifs, which in orthoretroviruses are irreplaceable for multitude functions including viral RNA genome recognition and packaging. Instead, FV Gag contains glycine-arginine-rich (GR) sequences at its C-terminus. In case of prototype FV (PFV) these are historically grouped in three boxes, which have been shown to play essential functions in genome reverse transcription, virion infectivity and particle morphogenesis. Additional functions for RNA packaging and Pol encapsidation were suggested, but have not been conclusively addressed.

**Results:**

Here we show that released wild type PFV particles, like orthoretroviruses, contain various cellular RNAs in addition to viral genome. Unlike orthoretroviruses, the content of selected cellular RNAs in capsids of PFV vector particles was not altered by viral genome encapsidation. Deletion of individual GR boxes had only minor negative effects (2 to 4-fold) on viral and cellular RNA encapsidation over a wide range of cellular Gag to viral genome ratios examined. Only the concurrent deletion of all three PFV Gag GR boxes, or the substitution of multiple arginine residues residing in the C-terminal GR box region by alanine, abolished both viral and cellular RNA encapsidation (>50 to >3,000-fold reduced), independent of the viral production system used. Consequently, those mutants also lacked detectable amounts of encapsidated Pol and were non-infectious. In contrast, particle release was reduced to a much lower extent (3 to 20-fold).

**Conclusions:**

Taken together, our data provides the first identification of a full-length PFV Gag mutant devoid in genome packaging and the first report of cellular RNA encapsidation into PFV particles. Our results suggest that the cooperative action of C-terminal clustered positively charged residues, present in all FV Gag proteins, is the main viral protein determinant for viral and cellular RNA encapsidation. The viral genome independent efficiency of cellular RNA encapsidation suggests differential packaging mechanisms for both types of RNAs. Finally, this study indicates that analogous to orthoretroviruses, Gag – nucleic acid interactions are required for FV capsid assembly and efficient particle release.

**Electronic supplementary material:**

The online version of this article (doi:10.1186/s12977-014-0087-7) contains supplementary material, which is available to authorized users.

## Background

Gag is the main structural protein of retroviruses that orchestrates the highly regulated process of particle assembly (reviewed in [1]). In addition to its function in assembly, Gag also secures the selective packaging of dimeric viral genomic RNAs (vgRNAs) from a cytoplasmic pool that consists of a substantial excess of non-viral and spliced viral RNAs (reviewed in [2-5]).

Orthoretroviral Gag proteins mediate the interaction with nucleic acids largely but not entirely by the C-terminal nucleocapsid (NC) domain (reviewed in [5]). They contain conserved motifs of regularly spaced cysteine and histidine residues (Cis-His), which are important for recognition of the complex structured packaging signal (called Psi) in the vgRNA [6]. This specific mechanism allows selective vgRNA encapsidation in orthoretroviral particles (reviewed in [2,7]). However, orthoretroviral Gag proteins also possess additional non-specific RNA binding features and several types of retroviruses were found to encapsidate cellular RNAs constituting up to 50% of the particle-associated nucleic acid content [8]. Most non-viral RNAs are packaged in a non-selective fashion, although in some cases a selective encapsidation is observed leading to their enrichment similar to vgRNA. The potential biological role of encapsidated host-cell RNAs for viral replication is largely unknown. Besides its participation in recognition and encapsidation of vgRNA the orthoretroviral NC domain has nucleic acid chaperone activities as well as additional functions in particle assembly and release, and timing of reverse transcription (RTr) (reviewed in [2,9]).

Hepatitis B virus (HBV), another virus encapsidating a RNA genome, which undergoes reverse transcription and shares several features of its replication cycle with FVs, seems to have another mechanism of RNA packaging. It encodes a core protein with N-terminal assembly and C-terminal nucleic acid binding domain, the latter being strongly enriched in basic amino acids (reviewed in [10]). In contrast to orthoretroviruses, encapsidation of HBV pregenomic RNA (pgRNA) requires HBV polymerase (P-protein) coexpression, preferentially in cis, and P-protein binding to a 5′ RNA stem loop structure within the pgRNA molecule [11,12]. However, in vivo the specificity of pgRNA packaging is further influenced by the phosphorylation status of the core protein, although it was demonstrated that it possesses phosphorylation-independent unspecific RNA binding activities in vitro [13,14]. Whether released HBV particles also contain encapsidated cellular RNAs has not been investigated to date.

FVs or spumaviruses constitute the only genus in a separate subfamily of retroviruses, the *spumaretrovirinae*. This is because FVs, although closely related to orthoretroviruses show several unique features in their replication strategy, some of which resemble features of HBV (reviewed in [15]). For example, unlike orthoretroviruses the FV Gag proteins are not processed into the canonical matrix (MA), capsid (CA) and NC subunits during particle morphogenesis. FV particles, which are released in an FV Env-dependent manner [16], contain a capsid of immature morphology consisting of Gag precursor (p71^Gag^ for PFV) and a large processing product (p68^Gag^ for PFV) that is derived from the precursor by proteolytic processing through the viral protease at a single cleavage site. Furthermore, FV Gag proteins lack the characteristic orthoretroviral Cys-His motifs [17]. It is believed that glycine-arginine-rich (GR) motifs in the FV Gag C-terminus represent a functional equivalent of the orthoretroviral Cys-His motifs (reviewed in [18]). Indeed, others and we have previously shown that these FV Gag motifs, which are grouped in three GR boxes (GRI-III) in PFV Gag, have essential functions in genome RTr, virion infectivity as well as particle morphogenesis [19,20]. However, up to date it remains unclear if individual FV GR motifs also mediate RNA packaging. Initial studies proposed for the PFV Gag GR box I (GRI) similar functions in the selective encapsidation of vgRNAs as assigned to retroviral NC domains and their Cys-His motifs [19,21]. Other studies failed to confirm a role of individual PFV Gag GR boxes for RNA packaging and only PFV Gag proteins with large C-terminal truncations lacking all GR boxes were reported to have lost vgRNA packaging capacity [20,22,23]. Further controversy arises from the fact that only the different simian FV (SFV) Gag proteins and PFV Gag contain clustered GR boxes, while no GR boxes were assigned for the non-primate FV Gag proteins [24,25]. However in general, all FV Gag proteins contain a high proportion of glycine and arginine residues in their C-termini. Unfortunately, only for PFV their function in viral replication was experimentally examined to date.

Similarly debated is the question of how FVs encapsidate their Pol protein, which is translated as a separate protein and not as an orthoretroviral-like Gag-Pol fusion protein. It is hypothesized that Gag as well as the Pol precursor both bind to vgRNA, where the RNA serves as a bridging molecule for Pol encapsidation [19,20,22,26-28]. However, another study challenged this view and instead proposed Pol encapsidation to require an accessory, direct Gag-Pol protein interaction involving positively charged residues of GRI [23].

Thus, despite being addressed in several studies, the determinants for FV RNA packaging and Pol encapsidation still remain unclear. As previous studies indicated that not an individual GR box motif mediates FV RNA packaging [20,22,23], we hypothesized that RNA packaging might be a cooperative function of the whole FV Gag C-terminus and in particular the arginine residues therein. We therefore, adopted a more “global” GR-box view and analyzed the combined role of positively charged arginine residues within the GRI-III boxes of PFV Gag in viral replication. We find that the arginine residues in the PFV Gag C-terminus cooperatively mediate viral and cellular RNA encapsidation and that Gag – nucleic acid interactions are required for capsid assembly, efficient particle release as well as Pol precursor packaging.

## Results

### Minor contribution of individual GR boxes for vgRNA encapsidation

An initial study suggested GRI as the main determinant for PFV vgRNA packaging [19]. Using an extensive, structured analysis comprising all three individual PFV Gag GR boxes mutated separately we recently highlighted novel GR box functions in RTr and particle morphogenesis [20]. In this study, which employed an expression-optimized 4-component PFV vector system, we found only minor contributions of individual GR boxes for functions in vgRNA packaging. To fully exclude that effects of individual GR boxes on vgRNA packaging were missed due to the rather high concentration of the provided transfer vector, we re-evaluated the potential contribution of individual GR boxes for packaging of vgRNA using an optimized qPCR setup and additionally extended the analysis to also include conditions of limited availability of packageable virus genome. To avoid any influence of the differential intra-particle RTr capacities of the individual GR box Gag mutants on the quantitative analysis [20] we used an RT-deficient PFV Pol packaging construct for the production of all particle samples.

**Figure 1:**
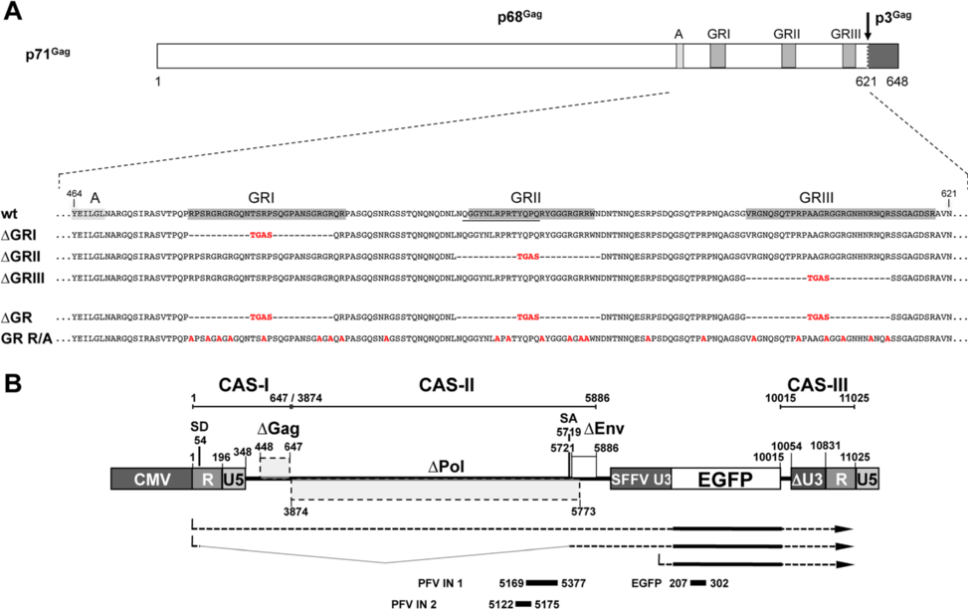
**PFV Gag mutants used in the study. (A)** Schematic outline of the PFV Gag precursor protein. A solid arrow marks the primary processing site in p71^Gag^. The enlargement shows the protein sequences of the C-termini of wild type PFV Gag (wt) p68^Gag^ subunit and the established GR box deletion mutant (∆GR) as well as the mutant having 23 C-terminal arginine residues substituted by alanine (GR R/A). Amino acid residues introduced to or altered in comparison to the wild type sequence are indicated in red bold typeset. Differentially shaded boxes highlight C-terminal structural and functional domains. A, assembly domain; GRI-III, glycine-arginine-rich boxes I to III. The described minimal chromatin-binding sequence is underlined [32,33]. The numbers indicate the amino acid position in the wt Gag protein. **(B)** Schematic outline of the PFV transfer vector puc2MD9 used. CAS: cis acting sequences I to III required for productive transduction. CMV: cytomegalovirus immediate early promoter, R: long terminal repeat (LTR) repeat region; U5: LTR unique 5′ region; ∆U3: enhancer – promoter deleted LTR unique 3’ region; partial coding sequences of PFV Gag, Pol and Env overlapping the CAS sequences are indicated by dashed boxes and marked with ∆. SFFV U3: spleen focus forming virus U3 enhancer promoter; EGFP: enhanced green fluorescent protein ORF; numbers indicate nucleotide positions in the HSRV2 RNA genome. Below the schematic outline the amplicons generated by the qPCR primer – probes sets (Table 1) specific for PFV *pol* or *egfp* ORFs are shown. Numbers indicate nucleotide position in the HSRV2 RNA genome or the *egfp* ORF.

**Figure 2:**
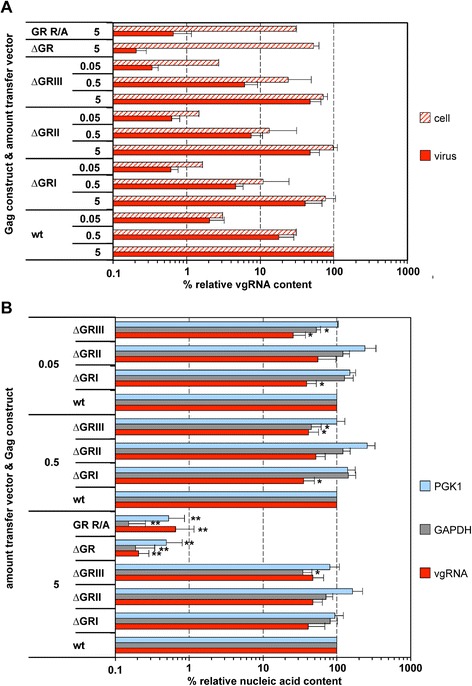
**Viral and non-viral RNA encapsidation by Gag mutants with individual GR box deletions.** 293T cells were co-transfected with puc2MD9, pcoPP, pcoPE and either pcoPG4 (wt), pcoPG ∆GRI (∆GRI), pcoPG ∆GRII (∆GRII), pcoPG ∆GRIII (∆GRIII), pcoPG4 ∆GR (∆GR), or pcoPG4 GR R/A (GR R/A). The amount of packaging constructs was kept constant whereas different amounts of transfer vector puc2MD9 (5, 0.5, 0.05 μg) were used as indicated. The total amount of DNA used for transfection was kept constant by filling up with pUC19. Subsequently extracted particle-associated nucleic acids and total cellular RNA samples were subjected to qPCR analysis using different primer-probe sets as summarized in Table 1. **(A)** Cellular and viral particle-associated levels of vgRNA. Viral particle and cellular nucleic acid content was determined by qPCR using specific primer – probes sets for PFV Pol. Mean values and standard deviation (n = 3-6) are shown as relative values compared to the wild type control. Viral particle values were normalized for Gag content, cellular values were normalized per ng of total RNA. **(B)** Viral particle-associated levels of vgRNA and selected cellular mRNAs. Viral nucleic acid content was determined by qPCR using specific primer – probes sets for PFV Pol, as well as human PGK1 and GAPDH mRNAs. Mean values and standard deviation (n = 3-6) are shown as relative values compared to the wild type control in each set of transfections varying in their amount of transfer vector as indicated. Viral particle values were normalized for Gag content. Differences between means of the wild type and the individual mutants were analyzed by Welch’s *t* test (*, p < 0.05; **, p < 0.01).

We characterized the nucleic acid composition of three sets of wild type (wt) and mutant PFV particles harboring individual GR box deletions (∆GRI, ∆GRII, ∆GRIII) (Figure 1A), generated by transfecting cells with different amounts of transfer vector, ranging over three orders of magnitude (Figure 2). The amount of Gag, Pol and Env packaging constructs was kept constant in all samples, which resulted in a similar virus production and particle release in all samples ([20], data not shown).

For particles containing wild type Gag a good correlation between the amount of transfer vector, the cell-associated (normalized for total RNA), and the particle-associated vgRNA copies (normalized for Gag content) was observed (Figure 2A, wt). In contrast to wild type, particles derived from Gag proteins with individual GR box deletions showed a slightly reduced (~2-fold) vgRNA encapsidation capacity relative to the corresponding cellular levels (Figure 2A, ∆GRI, ∆GRII, ∆GRIII). Only ∆GRIII mutant particles showed an up to 8-fold reduced relative encapsidation capacity at the lowest amount of transfer vector (Figure 2A, ∆GRIII 0.05).

The differences in relative vgRNA content (normalized for Gag content) of the various mutant particles in comparison to wild type became more apparent when each sample set (varying in their amount of transfer vector) was examined separately (Figure 2B). Independently of the amount of transfer vector, all individual GR box deletions showed only a very moderate (2 to 4-fold) reduction in vgRNA encapsidation (Figure 2B, red bars). The differences to wild type were statistical significant (p < 0.05) for ∆GRI and ∆GRIII samples in the 0.5 μg and 0.05 μg transfer vector group.

In summary, these results demonstrate that individual GR box sequences contribute only moderately to viral genome encapsidation, independently of the ratio of capsid to vgRNA present in the cell during particle assembly. This is in line with our previous findings at even higher cellular concentrations of packageable vgRNA [20]. Thus, we are unable to reproduce the reported major role of GRI for PFV vgRNA packaging [19].

### Global mutants in the PFV Gag GR-rich C-terminal domain display reduced particle release capacity and are non-infectious

As individual GR box motifs contribute only very little to viral genome encapsidation, even under conditions of limited availability of packageable vgRNA, we hypothesized that instead the whole Gag C-terminus, and the positively charged residues in particular, might mediate RNA encapsidation. In line with this hypothesis, non-simian FVs do contain a high number of arginine residues in their C-terminus, but lack the typical primate FV clustering into GR boxes [24,25]. Furthermore, Lee et al. reported positively charged residues as the main determinants of GRI box function in Pol encapsidation [23]. To examine the functional role of GR-rich sequences in the PFV Gag C-terminus for vgRNA encapsidation we generated packaging constructs for two “global” PFV Gag GR-box mutants (Figure 1A). One mutant (∆GR) has all three PFV Gag GR boxes deleted whereas in a second (GR R/A) 23 arginine residues in the C-terminal part of Gag containing GRI to GRIII were substituted by alanine.

**Figure 3:**
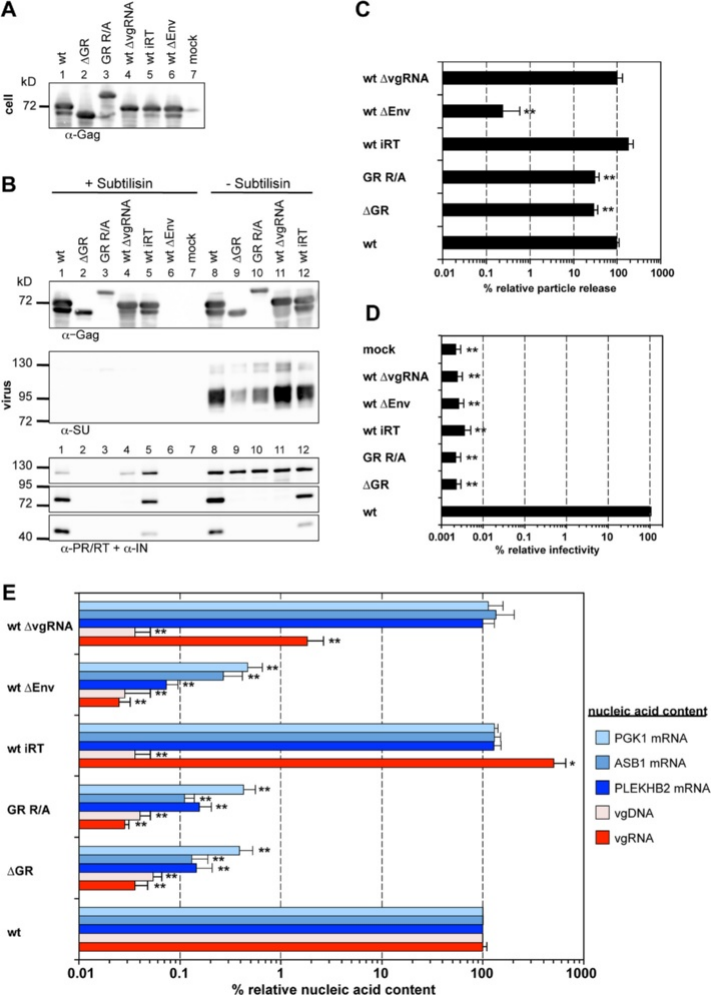
**Analysis of the GR-rich PFV Gag C-terminus in context of a replication-deficient vector system.** 293T cells were co-transfected with pMD9, pcziPol, pcoPE and either pcoPG4 (wt), pcoPG4 ∆GR (∆GR), or pcoPG4 GR R/A (GR R/A). As controls, cells were transfected with pcoPE, pcoPG4 and pcziPol (wt ∆vgRNA), with pMD9, pcoPE, pcoPG4 and pcziPol iRT (wt iRT), with pMD9, pcoPG4 and pcziPol (wt ∆Env) or only with pcDNA3.1 zeo + (mock). **(A, B)** Representative Western blot analysis of viral particles (virus) purified from 293T cell culture supernatant by ultracentrifugation through 20% sucrose and 293T cell lysates (cell). PFV proteins were detected using antibodies specific for PFV Gag (α-Gag), for PFV Pol PR/RT and IN (α-PR/RT + α-IN), or for PFV Env SU (α-SU). **(C)** Viral particle release was determined by quantitative Western blot analysis of viral particles. Mean values and standard deviations (n = 3-6) are shown as relative values compared to the wild type control and normalized for cellular expression levels. **(D)** Infectivity analysis of PFV particle containing cell culture supernatants. The values obtained using wild type PFV Gag expression plasmids were arbitrarily set to 100%. Relative means and standard deviations from six independent experiments are shown. Absolute titers of wt supernatants ranged between 2 × 10^6^ and 1.3 × 10^7^ eGFP ffu/ml. **(E)** Viral particle nucleic acid content was determined by qPCR using specific primer – probes sets for PFV Pol, as well as human PGK1, ASB1 and PLEKHB2 mRNAs as summarized in Table 1. Mean values and standard deviation (n = 3-6) are shown as relative values compared to the wild type control. Values were not normalized for Gag content. Differences between means of the wild type and the individual mutants in **(C)**-**(E)** were analyzed by Welch’s *t* test (*, p < 0.05; **, p < 0.01).

In order to avoid potential secondary effects of the introduced mutations on vgRNA structure and the expression pattern of the viral proteins we used a 4-component PFV vector system to characterize the mutant Gag phenotype in the first place. When 293T cells were co-transfected with transfer vector as well as packaging constructs for Env, Pol and the respective Gag proteins, both Gag mutants were expressed at wild type levels and released as viral particles, albeit in significantly reduced amounts compared to wt (3 to 4-fold) (Figure 3A-C). We did not observe mutant Gag processing neither in the cell lysates (Figure 3A, lane 2, 3) nor in the viral supernatants (Figure 3B, lane 2, 3, 9, 10). Interestingly, both mutant Gag proteins (∆GR and GR R/A) migrated higher than their expected molecular weight, a phenomenon we previously observed already for Gag ∆GRII mutants (Figure 3A + B, lane 2, 3, 9, 10) [20]. The released Gag protein was associated with Env and the relative amounts of both proteins correlated well (Figure 3B, lane 9, 10). The subtilisin resistance of Gag but not Env SU in the particle samples indicated that mutant Gag is released as membrane enveloped particulate material and not as free protein (Figure 3B) [29].

In contrast to Gag and Env, no mature Pol subunits (>50-fold reduced) were detectable in particle preparations of the Gag ∆GR and GR R/A mutants. Only released Pol precursor was detected in the samples, which was readily digested by subtilisin and was therefore not particle-associated (Figure 3B, lane 2, 3, 9, 10) as demonstrated previously [30].

In line with the Pol encapsidation and Gag processing defect of the PFV Gag ∆GR and GR R/A particles, we could not detect any infectivity of the respective viral supernatants (Figure 3D). Thus, the PFV Gag ∆GR and GR R/A mutant show at least a 10,000-fold decrease in virion infectivity, although their particle release was diminished only 3 to 4-fold (Figure 3C + D).

**Figure 4:**
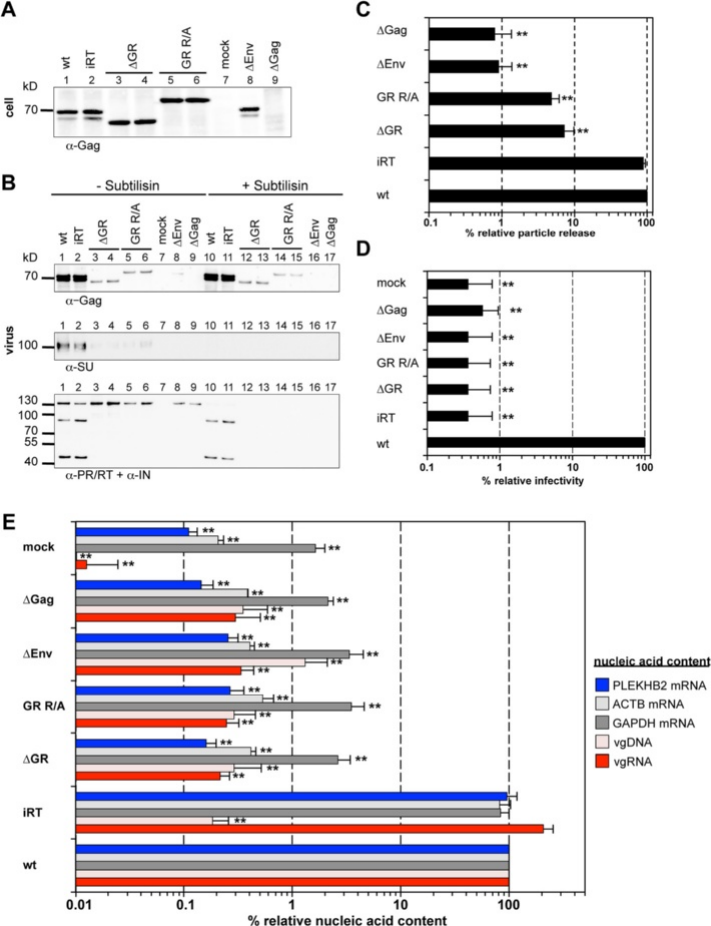
**Analysis of the GR-rich PFV Gag C-terminus in context of replication-competent proviral expression constructs.** 293T cells were transfected with pczHSRV2 (wt), pczHSRV2 ∆GR (∆GR), or pczHSRV2 GR R/A (GR R/A). As controls, cells were transfected with variants pczHSRV2 iRT (iRT), expressing a Pol protein with enzymatically inactive RT domain; pczHSRV2 iEnv (∆Env), with inactivated Env translation start; pczHSRV2 M78 (∆Gag), with inactivated Gag translation start; or only with pUC19 (mock). **(A, B)** Representative Western blot analysis of viral particles (virus) and 293T cell lysates (cell). PFV proteins were detected using antibodies specific for PFV Gag (α-Gag), for PFV Pol PR/RT and IN (α-PR/RT + α-IN), or for PFV Env SU (α-SU). **(C)** Viral particle release was determined by quantitative Western blot analysis of viral particles. Mean values and standard deviations (n = 3-6) are shown as relative values compared to the wild type control and normalized for cellular expression levels. **(D)** Infectivity analysis of PFV particle-containing cell culture supernatants. The values obtained using wild type PFV proviral expression plasmids were arbitrarily set to 100%. Relative means and standard deviations from three independent experiments are shown. Absolute titers of wt supernatants ranged between 6 × 10^3^ and 7 × 10^4^ eGFP ffu/ml. **(E)** Viral particle nucleic acid content was determined by qPCR using specific primer – probes sets for PFV Pol, as well as human ACTB, GAPDH and PLEKHB2 mRNAs as summarized in Table 1. Mean values and standard deviation (n = 4-6) are shown as relative values compared to the wild type control. Values were not normalized for Gag content. Differences between means of the wild type and the individual mutants in **(C)**-**(E)** were analyzed by Welch’s *t* test (**, p < 0.01).

Both Gag mutants were also examined in context of proviral expression constructs with sequences of authentic codon-usage to ensure that the observed phenotypes are not due to potentially altered uncoupled viral expression generated by the 4-component PFV vector system (Figure 4). In general the proviral expression system displays a roughly 10-fold lower wild type particle release than achieved using the 4-component vector system [31]. Overall the analysis revealed a similar phenotype in respect to Gag processing, Pol incorporation and viral infectivity for both Gag mutations (Figure 4A-D). Only their particle release deficiency was more pronounced in comparison to the 4-component vector system as particle-associated Gag levels were reduced up to 20-fold in comparison to wild type (Figure 4B + C). Of note Western blot analysis of particle-associated mature Pol proteins in samples generated with the proviral expression constructs shown in Figure 4B was close to the detection limit due to the reduced particle release of this system compared to the 4-component vector system [31]. Nevertheless, using larger amounts proviral construct derived, pelleted viral particles mature IN subunits were detectable in 20-fold diluted wt samples but were absent in virion samples of both mutants ∆GR and GR R/A (see Additional file 1).

In summary, the removal of all PFV Gag GR boxes or replacement of their residing arginine residues results in a reduced release of particles that are non-infectious and lack any detectable amounts of encapsidated Pol proteins.

### Positively charged amino acids of the PFV Gag GR-rich motifs are essential for efficient viral genome encapsidation

To characterize the viral genome encapsidation features of the two global Gag GR box mutants, the nucleic acid composition of wild type and mutant particles as well as several controls was determined by qPCR using different primer – probe sets specific for viral vector genome (Figure 1B, Table 1).

**Table 1:** **qPCR Primer/probe sets**

**Target**	**Primer/Probe**	**5′-3′ sequence** ^**a**^	**Cycle conditions**
PFV genome (Integrase 1)	fwd	CTTCAACCTTTGCTGAATG	95°C, 8 min, 1×
	rev	TAATACAGGGCTATAGGTGT	95°C, 30 s, 40×
	probe	FAM-TTGGAATTCAGTACTCCTTATCACCC-BHQ1	58°C, 30 s, 40×
PFV genome (Integrase 2)	fwd	TGCAATTCCAAAGGTGATTC	95°C, 8 min, 1×
	rev	TACCTCTTTCCTTTGCCCAT	95°C, 30 s, 40×
	probe	FAM-TCAAGGTGCAGCATTCACTTCTTCAA-BHQ1	59°C, 30 s, 40×
EGFP	fwd	GCAGTGCTTCAGCCGCTAC	95°C, 8 min, 1×
	rev	AAGAAGATGGTGCGCTCCTG	95°C, 30 s, 40×
	probe	HEX-CCGACCACATGAAGCAGCACGACTT-BHQ2	59°C, 30 s, 40×
			72°C, 45 s, 40×
GAPDH	fwd	CATCAATGGAAATCCCATCA	95°C, 45 s, 40×
	rev	GACTCCACGACGTACTCAGC	95°C, 30 s, 40×
	probe	FAM-TCCAGGAGCGAGATCCCTCCA-BHQ1	59°C, 30 s, 40×
			72°C, 30 s, 40×
ACTB	fwd	TGGACTTCGAGCAAGAGATG	95°C, 8 min, 1×
	rev	GAAGGAAGGCTGGAAGAGTG	95°C, 30 s, 40×
	probe	FAM-CGGCTGCTTCCAGCTCCTCC-BHQ1	59°C, 30 s, 40×
			72°C, 30 s, 40×
ASB1	primer/probe mix	HS00211548_m1 Gene Expression Kit Applied Biosystems	95°C, 10 min, 1×
			95°C, 15 s, 40×
			60°C, 1 min, 40×
PGK1	primer/probe mix	HS99999906_m1 Gene Expression Kit Applied Biosystems	95°C, 1 min, 1×
			95°C, 15 s, 40×
			60°C, 1 min, 40×
PLEKHB2	primer/probe mix	HS00215820_m1 Gene Expression Kit Applied Biosystems	95°C, 10 min, 40×
			95°C, 15 s, 40×
			60°C, 1 min, 40×

^a^Fam: 6-carboxyfluorescein; HEX: hexachloro-fluorescein; BHQ1: Black Hole Quencher 1; BHQ2: Black Hole Quencher 2.

Consistent with the infectivity defect of the PFV Gag ∆GR or GR R/A mutants, we were unable to detect any particle-associated viral genomic nucleic acids (vgRNA, vgDNA) above background levels (>3,000-fold reduction) in respective particle preparations generated by the 4-component vector system, neither when using primers and probes specific to PFV *pol* (Figure 3E, compare ‘∆GR’ and ‘GR R/A’ to ‘wt ∆Env’ and ‘wt’) nor when using primers specific to the *egfp* ORF (data not shown). This indicated a functional defect at the level of vgRNA encapsidation for both mutants. Again a similar analysis of both Gag mutants in context of proviral expression constructs revealed an identical phenotype in respect to viral genome encapsidation (Figure 4E).

These results strongly suggest that the cooperative action of clustered positively charged amino acids in the Gag C-terminus rather than individual GR-box motifs is essential for viral genome encapsidation.

### PFV particles encapsidate cellular RNAs in a Gag GR-rich motif-dependent manner

Interestingly, our analysis of particle-associated nucleic acids indicated that wild type FV particles can encapsidate low levels (2% of wt) of subgenomic viral RNAs containing the authentic *pol* ORF (detected by the *pol*-specific qPCR, Figure 1B) in the absence of vgRNA (Figure 3E, wt ∆vgRNA). However, this Pol packaging construct-derived RNA was not reverse transcribed since at the same time no vDNA was detectable (Figure 3E, wt ∆vgRNA). As other retroviruses are known to encapsidate subgenomic vRNAs and cellular RNAs by different mechanisms in addition to the viral genome [8], it seemed likely, although not previously examined, that FVs might also encapsidate cellular RNAs. Furthermore, since our Gag ∆GR and GR R/A mutants showed no detectable vgRNA packaging we were intrigued by the question if they might package cellular RNAs or if the mutations abolished RNA encapsidation in general.

We therefore examined the encapsidation of cellular RNAs into released wild type and mutant FV particles. For this purpose we determined by qPCR the presence of selected RNA species, some of which were reported previously to be packaged into murine leukemia virus (MLV) or human immunodeficiency virus (HIV) particles [8], in nucleic acid preparations of different PFV particle samples generated using the 4-component vector system or proviral expression constructs (Figure 3E + Figure 4E). Relative copy numbers of cellular PGK1, ASB1, PLEKHB2, ACTB and GAPDH mRNAs of wild type PFV particle preparations were at least 50 to 1,000-fold higher than those of mock particle preparations derived from 293T cells transfected with pUC19 (mock) or particle preparations of samples not expressing PFV Env (∆Env) (Figure 3E + Figure 4E). Our results show for the first time that FVs can also package cellular RNAs. Interestingly, the level of the cellular mRNAs in PFV particles was not significantly influenced by the presence or absence of vgRNA (Figure 3E, compare ‘wt’ and ‘wt iRT’ to ‘wt ∆vgRNA’). Most importantly however, we were unable to detect those cellular RNAs in Gag ∆GR and GR R/A derived particle preparations (Figure 3E + Figure 4E, compare ‘∆GR’ and ‘GR R/A’ to ‘wt’) indicating that these mutants lost RNA packaging capacity in general.

This observation led us to also analyze the cellular RNA content of mutant particles with individual GR box deletions, since specific GR boxes may convey cellular RNA encapsidation. In contrast to the “global” PFV Gag GR-box mutants ∆GR and GR R/A, particle-associated cellular mRNA contents (PGK1, GAPDH) of particles with individual GR box deletions was within a 2 to 3-fold range of wild type and not influenced by the amount encapsidated vgRNA (Figure 2B). Although below statistical significance, GRII deleted particles consistently showed a slightly increased PGK1 mRNA content (Figure 2B, light blue bars). Furthermore, ∆GRIII box deleted particles consistently showed the lowest content in GAPDH mRNA of all mutants that was statistically different to wild type at all three amounts of transfer vector used (p < 0.05) (Figure 2B, grey bars).

In summary, our analyses demonstrate that PFV wt Gag particles also contain cellular mRNAs. Furthermore, they show that the GR-rich sequences of PFV Gag but not individual GR-box motifs are essential for both viral and non-viral RNA encapsidation.

### PFV Gag GR-rich motif mutants lack chromatin tethering functions

**Figure 5:**
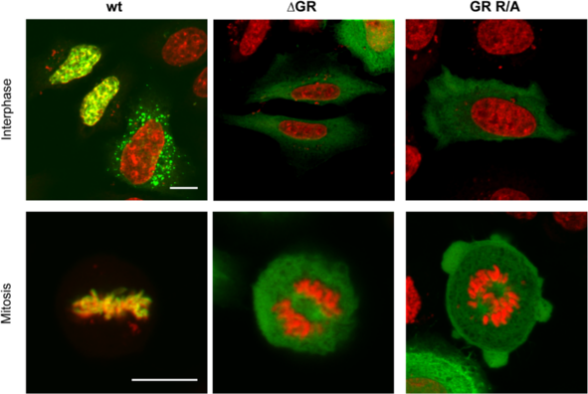
**Altered intracellular distribution of mutant Gag proteins.** Intracellular distribution of C-terminal eYFP-tagged Gag wt (wt), Gag ∆GR (∆GR) or Gag GR R/A (GR R/A) proteins in HeLa cells 48 h post transfection. Samples were fixed and stained with DAPI. The images show a merged image of the eYFP (green) and the DAPI (red) channel. Scale bar: 10 μm.

Sequences of GRII were shown to be involved in tethering PFV Gag to the host cell chromatin during mitosis [32,33]. We therefore examined the intracellular distribution of the two “global” PFV Gag GR-box mutants by confocal fluorescence microscopy of cells transfected with C-terminally EYFP tagged Gag variants. In contrast to wt Gag, PFV Gag ∆GR protein did not colocalize with host cell chromatin during mitosis (Figure 5, compare left and middle panels), showing the same phenotype as reported previously for a PFV Gag ∆GRII mutant protein [20,32,33]. Interestingly, the PFV Gag GR R/A mutant displayed an identical phenotype as ∆GR (Figure 5, right panel) and ∆GRII [20], indicating that the C-terminal arginine residues contribute to chromatin binding, although only two of them (R_540_ and R_542_) are located in the minimal chromatin binding site (CBS) as characterized by Tobaly-Tapiero and colleagues [32]. The identical phenotype of these Gag mutants suggests that changing as little as two arginine residues in the PFV Gag CBS are sufficient to inactivate its chromatin tethering function. In line with this hypothesis, Schneider et al. [34] recently demonstrated in context of chimeric MLV Gag proteins an inactivation of the chromatin tethering function of an inserted minimal PFV Gag CBS by mutating its two arginine residues.

### PFV GR box mutant particles display capsid assembly defects

**Figure 6:**
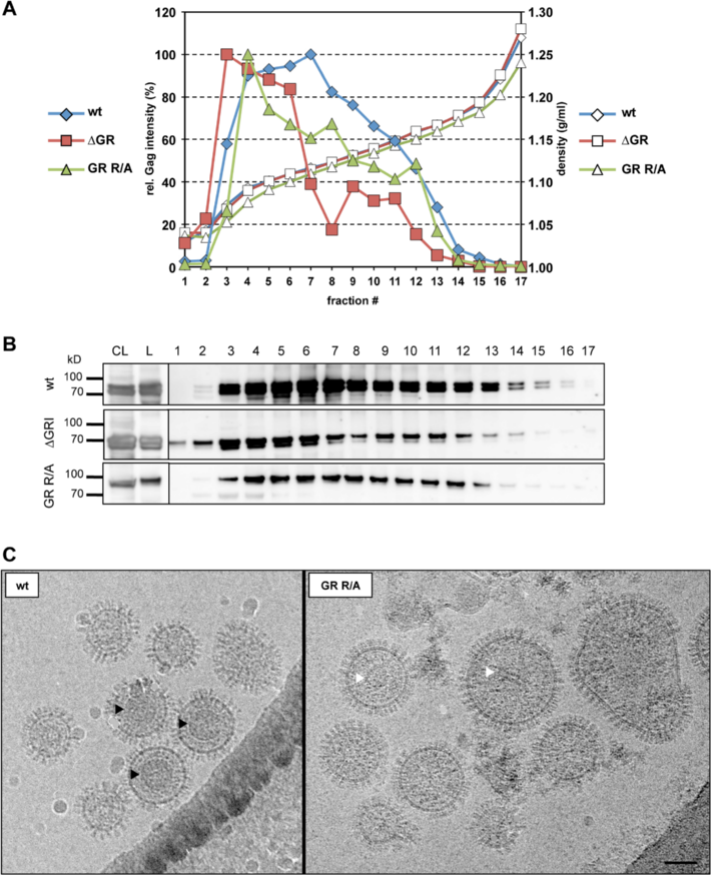
**Buoyant density and electron microscopy analysis of the mutant PFV particles.** 293T cells were co-transfected with either pcoPG4 (wt), pcoPG4 ∆GR (∆GR), or pcoPG4 GR R/A (GR R/A) in combination with pcoPP, pcoPE and puc2MD9 to yield the respective wt and mutant particles. **(A)** Concentrated virus supernatant was loaded onto an iodixanol step gradient ranging from 15 to 40%. After centrifugation at 197,000 g for 3 h the gradient was split into 17 fractions and their density determined by refractometry measurements (right ordinate) and their Gag content examined by Western blot analysis (left ordinate). The highest Gag signal intensity in each gradient was set to 100%. **(B)** Western blot analysis of the Gag content in individual gradient fractions (1-17), pelleted virus particles (L) and cell lysates of transfected 293T cells (CL) using polyclonal antibodies specific for PFV Gag (α-Gag) (see above). **(C)** Cryo-electron microscopy analysis of wild type PFV (left) and of GR R/A mutant (right) particles. The virus particles were concentrated via a 20% sucrose gradient centrifugation step followed by size exclusion column filtration. Black arrowheads indicate regular Gag assemblies in the wild type virus and white arrows mark putatively aberrant Gag assemblies in the GR R/A mutant. Scale bar: 60 nm.

In vivo particle assembly of some orthoretroviruses (e.g. MLV), but not others (HIV), is strongly dependent on Gag – nucleic acid interactions (reviewed in [5]). To investigate whether FV capsid assembly might be influenced by Gag – nucleic acid interactions we performed isopycnic ultracentrifugation in order to compare the buoyant density of wild type and mutant viral particles (Figure 6A + B). The analysis revealed a different density profile of mutant viral particles in comparison to wild type. Fractions with the highest Gag signal ranged from 3 to 5 in mutant samples, whereas the Gag peak in the wt preparation was located between fraction 6 and 7. Hence, both mutants seem to have slightly lighter particle densities, indicating aberrant particle morphology.

This was further supported by cryo electron microscopy analysis. Virus particles were produced by transiently transfected 293T cells expressing the PFV Gag wild type or GR R/A mutant proteins in the context of the 4-component vector system and concentrated by ultracentrifugation followed by size exclusion filtration prior to electron microscopy analysis. Gag expression levels of wild type and Gag ∆GR or GR R/A expressing cells were comparable (Figure 3A) and particle release was readily detectable for both mutants, though at a reduced level (Figure 3B + C). Pronounced morphological differences of GR R/A mutant particles in comparison to wild type particles were detectable in cryo electron micrographs (Figure 6C). Unlike wild type PFV particles, which contained significant numbers of virions with a clearly visible regular shaped capsid structure (Figure 6C left, black arrowheads), no such structures were detectable in GR R/A particle preparations (Figure 6C right). In addition, GR R/A particles appeared to be more heterogeneous in size and some contained irregularly shaped electron dense material (Figure 6C right, white arrowheads).

Thus, PFV Gag – nucleic acid interactions seem to be important for correct capsid assembly that might be required for efficient particle release in vivo.

## Discussion

In this study we characterized the PFV Gag determinants essential for vgRNA encapsidation. We demonstrate that independent of the intracellular ratio of Gag to vgRNA the whole GR-rich Gag C-terminus rather than individual GR box motifs mediates vgRNA encapsidation. In line with our results Stenbak and colleagues reported that truncation of the whole Gag C-terminus comprising the GR-rich sequences and the p3 domain can abolish vgRNA packaging [22]. The “global” GR-box PFV Gag mutant GR R/A, having 23 arginine residues (out of 65 total arginine or lysine residues present in p71^Gag^) in the C-terminus of Gag replaced by alanine, is the first full-length PFV Gag mutant reported to have no vgRNA packaging capacity. Harboring “only” 23 amino acid changes, this mutant indicates that the clustering of positively charged residues is the main Gag determinant required for vgRNA encapsidation, independent of the viral production system (replication-deficient vectors vs. replication-competent proviruses) used for analysis. A general requirement of positively charged residue enrichment instead of their clustering into GR boxes as RNA-binding motifs can elegantly explain how non-primate FVs package vgRNA while lacking GR boxes. In line with this hypothesis the Gag C-termini of feline FV, equine FV, and bovine FV contain 20, 23, and 22 arginine residues respectively (reviewed in [18]).

We also report here for the first time the encapsidation of various cellular RNAs into PFV particles. Notably, unlike reported for MLV and HIV-1 we did not observe any significant difference in their copy numbers whether wild type PFV vector particles contained or lacked vgRNA [8]. This suggests that vgRNA and non-viral RNAs do not compete with each other, which might indicate different mechanisms of encapsidation of both types of RNA into PFV particles. Since the positively charged residues in the Gag C-terminus also appear to control packaging of all non-viral RNAs examined, the basis of selective encapsidation of vgRNA remains to be defined. In general, however, our results indicate that by not relying on specific Gag motifs FVs seem to have a fundamentally different RNA packaging strategy than other retroviruses. The analogy to Hepadnaviruses, which share some features in their replication strategy with FVs, may indicate that the specificity of vgRNA encapsidation is influenced by the FV Gag phosphorylation status [13,14]. However, except for cellular PFV Gag being phosphorylated predominantly at serine residues reported by Enssle and colleagues [35] neither specific phosphorylation sites of Gag have been identified nor the influence of the Gag phosphorylation status on viral replication studied.

Although we have not formally shown that both global Gag GR box mutants are completely devoid of nucleic acids the absence of all cellular mRNAs examined (five in total) suggests that this is indeed the case. Therefore another interesting task to be examined in the future is the determination of the repertoire and abundance of coding or non-coding, non-viral RNAs co-packaged into wild type FV virions on a global scale and its comparison to other retroviruses. The PFV Gag GR box mutants described here will be an important tool/control for this kind of analysis. Furthermore, elucidation of the potential roles of various particle-associated non-viral RNAs in the retroviral replication cycle is a field that has not been widely studied so far.

Together with a lack of viral genome packaging we did not detect particle-associated mature PFV Pol subunits in virus samples of PFV Gag ∆GR and GR R/A mutants generated by different expression systems. In line with the absence of particle-associated Pol no processing of particle-associated mutant Gag protein was observed. Thus, we find that Pol is not encapsidated if no RNA is packaged. While, Lee and colleagues previously suggested GRI as a direct Gag-Pol interaction motif [23], we showed that deletion or substitution of GRI alone does neither abolish Pol nor RNA packaging [20]. Although we cannot fully exclude accessory contributions of Gag-Pol protein interactions our data strongly implies that Pol packaging does not require direct Gag-Pol interaction but rather occurs through an essential RNA bridge [27].

In case of MLV and Rous sarcoma virus (RSV) the Gag NC domain is strictly required for particle production [36,37], while several studies showed that the arginine-rich C-terminus of PFV Gag is not absolutely required to assemble and release viral particles [22,38,39]. We observed release of the “global” GR-box Gag mutants ∆GR and GR R/A, however, at a lower level than wild type, with the extent of the reduction being influenced by the expression system used. Similarly, in HIV-1 certain mutations in the Cys-His motifs significantly reduce particle production due to impaired Gag multimerization [40,41]. Albeit retroviral Gag multimerization does not require binding of nucleic acids it is greatly facilitated by their presence [42-44]. The absence of regularly shaped capsid structures in electron micrographs of released GR R/A mutant particles, the larger heterogeneity in size and the altered density profiles of mutant particles are in line with assembly and/or oligomerization defects of PFV Gag proteins lacking nucleic acid binding capacity. Therefore, we like to suggest that also wild type PFV Gag multimerization is facilitated by interactions of Gag with nucleic acids thereby enhancing capsid assembly and particle release. However, in the absence of vgRNA its structural function in PFV particle assembly and egress seems to be complemented by non-viral RNAs, as wild type particles lacking vgRNA (wt ∆vgRNA) still contain all non-viral RNAs examined and show wild type-like capsid assembly and particle release. In contrast, efficient Pol packaging seems to be absolutely dependent on the presence of vgRNA.

As FV Gag proteins lack a membrane targeting signal virus egress is dependent on capsid interactions with the glycoprotein leader peptide [16,45-47]. These interactions might be strongly influenced by Gag oligomerization and capsid assembly and thereby alter the efficiency of particle export. Differential levels of Gag and Env expression by FV vector and proviral expression systems used in our study may modulate the interaction capacity of the mutant capsids with the glycoprotein and explain the differences in the particle release deficiency observed for both virus production systems.

## Conclusions

In summary, our data sheds light on the close connection of RNA packaging, Pol encapsidation, capsid assembly, and as previously shown RTr in FV replication. We characterized here the first full-length PFV Gag protein mutant deficient in vgRNA encapsidation independent of the virus production system used. Furthermore, we show for the first time that like their orthoretroviral cousins FVs also encapsidate various non-viral RNAs. More importantly, we demonstrate that the positively charged residues in the whole PFV Gag C-terminus act cooperatively to package vgRNA but also all cellular mRNAs examined, indicating an RNA packaging mechanism different to other retroviruses. The nucleic acid binding abilities of Gag influence PFV particle assembly and egress. While cellular RNAs seem to be able to complement functions of vgRNA for particle assembly and egress they cannot complement its function for Pol packaging. These findings on PFV Gag nucleic acid interactions and encapsidation also provide additional lines of evidence strongly supporting the current model of PFV Pol packaging by a mechanism involving simultaneous binding of Gag and Pol to vgRNA.

## Methods

### Cells and culture conditions

The human kidney cell line 293T [48], the human epithelium HeLa cell line [49] and the human fibrosarcoma cell line HT1080 [50] as well as the clonal variant HT1080 PLNE thereof [31] were cultivated in Dulbecco’s modified Eagle’s medium (DMEM) supplemented with 10% heat-inactivated fetal calf serum and antibiotics. HeLa cells used for confocal laser scanning microscopy were cultivated in phenol red free media.

### Recombinant plasmid DNAs

A 4-component PFV vector system, consisting of the expression-optimized packaging constructs pcoPG4 (PFV Gag), pcoPE (PFV Env), pcoPP (Pol) or its authentic ORF containing variant pcziPol (PFV Pol), and the enhanced green fluorescent protein (eGFP)-expressing PFV transfer vectors pMD9 or puc2MD9 (Figure 1B), has been described previously [20,26,29]. In some experiments previously described variants of the PFV Pol packaging construct, containing expression-optimized (pcoPP2) or authentic (pcziPol iRT) ORFs with catalytically inactive reverse transcriptase (Pol iRT, YVDD_312–315_GAAA mutation), were used [20,51].

All PFV Gag packaging constructs used in this study are based on the parental pcoPG4 vector or its C-terminal EYFP tagged variant pcoPG4 CeYFP [29]. The PFV Gag packaging constructs used in this study are depicted in Figure 1A. The packaging constructs encoding mutant Gag protein with deletion in individual GR boxes (pcoPG4 ∆GRI, pcoPG4 ∆GRII, pcoPG4 ∆GRIII) have been described previously [20]. Two additional PFV Gag packaging constructs were generated for this study. The ∆GR mutant (pcoPG4 ∆GR, pcoPG4 CeYFP ∆GR) has all three GR boxes simultaneously deleted and each replaced by a TGAS peptide sequences as found in the original individual GR box deletion mutants [20]. In the GR R/A mutant (pcoPG4 GR R/A, pcoPG4 CeYFP GR R/A) 23 arginine residues between aa 485 and 614 of PFV Gag were replaced by alanine.

The CMV-driven proviral expression vector pczHSRV2 (wt) and its variants pczHSRV2 M69 (iRT), expressing a Pol protein with enzymatically inactive RT domain (YVDD_312-315_GAAA mutation), and pczHSRV2 M78 (∆Gag), having the Gag translation start inactivated (M_1_L, ATG to TTG; S_3_Stop, TCA to TAA mutation) were described previously [51,52]. For this study the variants pczHSRV2 ∆GR (∆GR), having all three GR boxes deleted and each replaced by TGAS peptide sequences, pczHSRV2 GR R/A (GR R/A), having 23 arginine residues between aa 485 and 614 of PFV Gag replaced by alanine, and pczHSRV2 iEnv (∆Env) having the Env translation start inactivated (M_1_T, ATG to ACG, M_5_T, ATG to ACG; M_16_T, ATG to ACG mutation) were generated. All constructs were verified by sequencing analysis. Primer sequences and additional details are available upon request.

### Transfection and virus production

Cell culture supernatants containing recombinant viral particles were generated by transfection of 293T cells with the corresponding plasmids using polyethyleneimine (PEI) as described previously [20,31]. For subsequent Western blot analysis the supernatant generated by transient transfection was harvested, passed through a 0.45-μm filter and centrifuged at 4°C and 25,000 rpm for 3 h in a SW40 or SW28 rotor (Beckman) through a 20% sucrose cushion. The particulate material was resuspended in phosphate-buffered saline (PBS). For cryo electron microscopy analysis viral particles were produced in serum-free medium and a further concentration step using Amicon Ultra 0.5 ml 100 K Concentrators was included following the first concentration by ultracentrifugation through 20% sucrose similar as described recently [53].

### Subtilisin digest

Subtilisin treatment of concentrated particles was performed as previously described [30,54]. Briefly, half of each purified particle pellet resuspended in PBS was incubated in a digestion mix containing final concentrations of 1 mM CaCl_2_, 50 mM Tris-HCl pH 8.0 and 25 μg/ml subtilisin. The mock treated other half was incubated with the digestion mix including PBS instead of subtilisin. The digestion was stopped after 2 h at 37°C by adding phenylmethylsulfonylfluoride (PMSF) at a final concentration of 100 μg/ml to each reaction prior to addition of 2× sodium dodecyl sulfate (SDS) protein sample buffer (PPPC; 100 mM Tris-HCl [pH 6.8], 24% glycerol, 8% SDS, 2% dithiothreitol, 0.02% Coomassie blue G-250).

### Infectivity analysis

Transduction efficiency of recombinant, eGFP-expressing PFV vector particles by fluorescence reporter-gene transfer assay was analyzed as described previously [55]. Virus particles generated by use of proviral expression plasmids were titrated on HT1080 PLNE cells harboring a Tas-inducible nuclear *egfp* ORF in their genome as described previously [31]. All transduction experiments were performed at least three times. In each independent experiment the values obtained with the wild type construct pcoPG4 and pczHSRV2, respectively, were arbitrarily set to 100% and values obtained with other constructs were normalized as a percentage of the wild type values.

### Western blot analysis

Cells from a single transfected 100-mm cell culture dish were lysed in detergent-containing buffer and the lysates were subsequently centrifuged through a QIAshredder column (QIAGEN). Protein samples from cellular lysates or purified particulate material were separated by SDS-PAGE on a 10% polyacrylamide gel and analyzed by immunoblotting as described previously [16]. Polyclonal rabbit antisera specific for PFV Gag [56] or the amino acids (aa) 1 to 86 of the PFV Env leader peptide (LP), [16] as well as hybridoma supernatants specific for PFV RT (clone 15E10) or PFV integrase (IN) (clone 3E11) [57] were employed. After incubation with a horseradish peroxidase (HRP)-conjugated secondary antibody, the blots were developed with Immobilon Western HRP substrate. The chemiluminescence signal was digitally recorded using a LAS-3000 (Fujifilm) imager and quantified using ImageGauge (Fujifilm).

### Confocal microscopy

The analysis of the intracellular distribution of C-terminal eYFP-tagged Gag constructs using confocal microscopy was done as described previously [33]. Briefly, HeLa cells were plated at a concentration of 6 × 10^4^ cells per well on cover slips in 12-well plates one day before transfecting them with 0.1 μg Gag C-terminal eYFP fusion expression plasmid (pcoPG4 CeYFP or mutants thereof as indicated) using FuGENE HD transfection reagent (Roche). At 48 h post transfection the cells were washed with cold PBS, fixed with 3% paraformaldehyde, and the cell nuclei were stained with DAPI for 5 min. Finally the cells were covered with Mowiol. Confocal laser scanning images were obtained on a Zeiss LSM 510 using a Zeiss Apochromat 63×, NA 1.4 oil immersion objective. Fluorescence images were evaluated using ImageJ software.

### Quantitative PCR analysis

Preparation of particle samples for qPCR analysis was performed as previously described [20,54]. Furthermore, cellular nucleic acids were extracted by trypsinizing transiently transfected 293T cells of one 100-mm cell culture dish. After terminating the trypsinisation reaction by addition of 4 ml complete medium the homogenous cells suspension was pelleted at 300 g for 5 min. Subsequently, the supernatant was aspirated and cellular total RNA was extracted from the cell pellet with the RNeasy Mini kit (QIAGEN) according to the manufacturers protocol. This included the recommended addition of beta-mercaptoethanol to buffer RLT, the use of QIAshredder columns for cell homogenization and omission of the optional DNaseI digestion on the spin column. Reverse transcriptase reaction including DNaseI digest was essentially done as for the particle samples, using 250 ng of total RNA and 10 pmol oligo (dT)_30_ or random hexamer primers in a total volume of 40 μl. Finally, for qPCR analysis of RNA content 4.5 μl of each reverse transcriptase reaction was analyzed in duplicates in a total reaction volume of 25 μl using Maxima Probe qPCR Master Mix including ROX dye (Thermo Scientific) and a StepOnePlus (Applied Biosystems) quantitative PCR machine. Primers, Taqman probes and cycling conditions for specific quantification of PFV genome, EGFP, or human GAPDH and ACTB are summarized in Table 1. Cellular and particle-associated copy numbers of ASB1, PGK1 and PLEKHB2 mRNAs were determined using the Taqman Gene Expression Assay Kits (HS00211548_m1, HS99999906_m1, HS00215820_m1 respectively, from Applied Biosystems) according to the manufacturers manual. All values obtained were referred to a standard curve consisting of 10-fold serial dilutions of respective reference plasmids containing the target sequences (puc2MD9qP for viral genomic sequences and EGFP, pCR2.1-TOPO-GAPDH, pCR.2.1-TOPO-ACTB, pCR2.1-TOPO-ASB1, pCR2.1-TOPO-PGK1 and pCR2.1-TOPO-PLEKHB2 respectively). All sample values included were in the linear range of the standard curves with a span from 10 to 10^9^ copies. The values for the DNA or RNA content of viral particle samples obtained by the qPCR analysis are expressed as percentage of the wild type (generated by transfection of cells with pcoPG4, pcoPP/pcziPol, pcoPE and puc2MD9 or alternatively pczHSRV2).

Absolute copy numbers corresponding to 0.5 ml plain supernatant of wild type samples ranged from 3.4 × 10^5^ to 6.2 × 10^7^ with a detection limit of 1.2 × 10^3^ for viral genomic RNA, 2.3 × 10^6^ to 2.8 × 10^7^ with a detection limit of 1.2 × 10^3^ for viral genomic DNA, 7.0 × 10^5^ to 6.9 × 10^6^ with a detection limit of 1.8 × 10^3^ for GAPDH mRNA, 2.4 × 10^4^ to 1.3 × 10^5^ with a detection limit of 1.8 × 10^2^ for ASB1 mRNA, 2.3 × 10^5^ to 1.3 × 10^6^ with a detection limit of 1.8 × 10^3^ for PGK1 mRNA, 8.2 × 10^4^ to 4.6 × 10^5^ with a detection limit of 1.8 × 10^2^ for PLEKHB2 mRNA, and 8.0 × 10^6^ to 9.3 × 10^6^ with a detection limit of 1.1 × 10^2^ for ACTB. Whether the nucleic acid copy number values of the different particle samples were normalized for Gag content by quantitative Western blot analysis is mentioned in the legend of the individual figures. Determined RNA values of cellular samples were calculated as copies/ng total RNA and expressed as percentage of the wild type.

### Cryo-electron microscopy

Wild type PFV and the GR R/A mutant samples were both observed by cryo electron microscopy (cryo-EM) following the same procedure except that wild type PFV particles were first inactivated for at least 1 h in 4% paraformaldehyde before being processed. In summary, 4 μl of sample containing 10 nm gold beads was applied to Quantifoil holey carbon grid and the grid was plunge frozen in liquid ethane with a Vitrobot (FEI, the Netherlands). The frozen grid was transferred to a FEI F20 FEG cryo electron microscope. Images were recorded at a nominal magnification of 29,000 on a 4 k by 4 k Eagle CCD camera. The gold beads were computationally removed from the field of view shown in Figure 6 for clarity.

### Density centrifugation

Density centrifugation was essentially performed as described before [30]. Briefly, particle preparations were concentrated from 60 ml culture supernatant of 293T cells transiently co-transfected using the 4-component vector system and resuspended in 175 μl PBS. This virus concentrate was then overlaid onto a 1.8 ml iodixanol-PBS (OptiPrep®) gradient consisting of nine 220 μl layers ranging from 15% to 40%. Following ultracentrifugation at 197,000 g and 4°C for 3 h (TLS55 rotor; Beckman) 17× 127 μl fractions were collected from top to bottom. Subsequently, the density of the fractions was determined by refractometry, as well as the Gag protein content examined by Western blot analysis.

## Additional file

Additional file 1:
**Analysis of Pol particle encapsidation in context of proviral expression constructs using highly concentrated viral particle samples.** Particle preparations derived from supernatants of 293T cells transiently transfected with proviral expression constructs pczHSRV2 (wt), pczHSRV2 ∆GR (∆GR), pczHSRV2 GR R/A (GR R/A) or pczHSRV2 iEnv (∆Env). Concentrated viral particles were incubated with subtilisin in order to digest non-particle-associated Pol protein prior to sample lysis and subsequent Western blot analysis. Wild type particle samples were serially diluted (lane 1-4) and viral proteins in wildtype and mutant particle samples were detected with rabbit polyclonal antisera specific for PFV IN (α-IN) or PFV Gag (α-Gag).
