# Supplementary figures and images for: The cooperative function of arginine residues in the Prototype Foamy Virus Gag C-terminus mediates viral and cellular RNA encapsidation
Source: Retrovirology. 2014 Oct 8;11:87. doi: 10.1186/s12977-014-0087-7 (PMC4198681; doi:10.1186/s12977-014-0087-7)

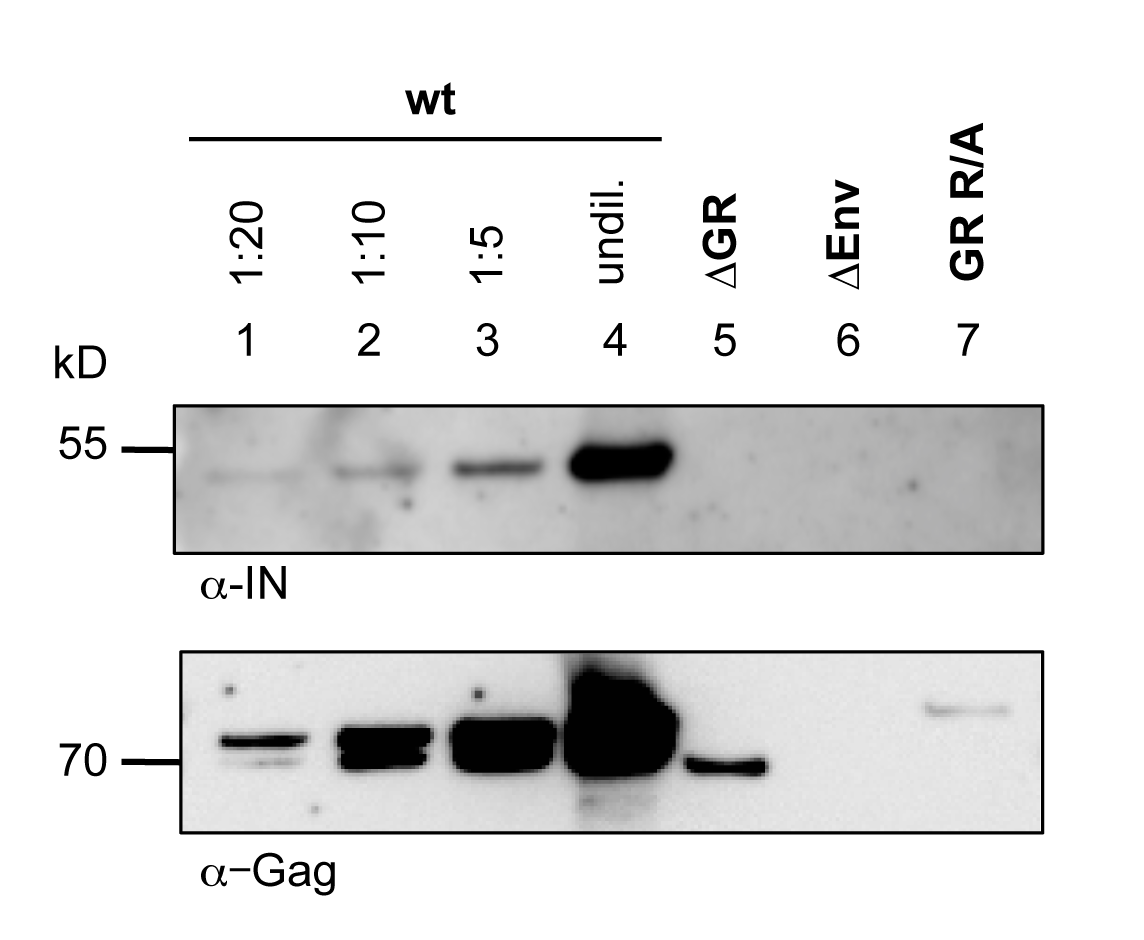

Supplement: Additional file 1: — Analysis of Pol particle encapsidation in context of proviral expression constructs using highly concentrated viral particle samples. Particle preparations derived from supernatants of 293T cells transiently transfected with proviral expression constructs pczHSRV2 (wt), pczHSRV2 ∆GR (∆GR), pczHSRV2 GR R/A (GR R/A) or pczHSRV2 iEnv (∆Env). Concentrated viral particles were incubated with subtilisin in order to digest non-particle-associated Pol protein prior to sample lysis and subsequent Western blot analysis. Wild type particle samples were serially diluted (lane 1-4) and viral proteins in wildtype and mutant particle samples were detected with rabbit polyclonal antisera specific for PFV IN (α-IN) or PFV Gag (α-Gag). [file 12977_2014_87_MOESM1_ESM.png]
